# Patient satisfaction with postoperative analgesia after hepatobiliary and pancreatic surgery: a retrospective analysis

**DOI:** 10.3389/fsurg.2026.1887173

**Published:** 2026-07-28

**Authors:** Wending Chen, Yeke Zhu, Xiaodong Tang, Xiangyang Cheng, Xuwu Xiang, Weiliu Zhu, Pin Wu, Canji Shou, Diansan Su, Shuyuan Gan

**Affiliations:** 1 Department of Anesthesiology, Women’s Hospital, Zhejiang University School of Medicine, Hangzhou, China; 2 Department of Anesthesiology, The First Affiliated Hospital, Zhejiang University School of Medicine, Hangzhou, China

**Keywords:** dezocine, hepatobiliary surgery, hyperhidrosis, patient satisfaction, patient-controlled analgesia, postoperative pain

## Abstract

**Objectives:**

Patient satisfaction is a key metric for evaluating healthcare quality. Patients undergoing hepatobiliary and pancreatic surgery frequently experience severe postoperative pain, necessitating patient-controlled intravenous analgesia (PCIA). This study aimed to identify factors associated with reduced satisfaction regarding PCIA in this context.

**Methods:**

A retrospective observational study analyzed data from adults receiving PCIA after elective hepatobiliary and pancreatic surgery. Univariate and multivariate logistic regression, employing a forced-entry method to adjust for a comprehensive panel of clinically relevant confounders [including age, sex, body mass index (BMI), American Society of Anesthesiologists (ASA) status, surgical trauma category, surgical approach, malignancy, and major postoperative complications], were used to examine associations between satisfaction and demographics, surgical details, analgesic regimens, postoperative pain (NRS), and adverse effects.

**Results:**

Among 1,667 patients, 108 (6.5%) were dissatisfied. Multivariable analysis identified moderate-to-severe pain (NRS > 3; aOR = 14.93, 95%CI: 9.52–26.32) and hyperhidrosis (aOR = 6.29, 95% CI: 3.05–13.89) as strong independent predictors of dissatisfaction (both *P* < 0.001). A significant interaction was found between dezocine and pain severity (*P* < 0.001). Among patients with NRS > 3, dezocine was associated with significantly lower odds of dissatisfaction (aOR = 0.18, 95% CI: 0.06–0.55), corresponding to an ∼87% reduction, whereas no significant benefit was observed in those with mild pain (aOR = 1.22, 95% CI: 0.33–4.68, *P* = 0.771). Hyperhidrosis was independently associated with rescue analgesia (OR = 8.82, *P* = 0.016).

**Conclusions:**

Dissatisfaction is driven by moderate-to-severe pain and hyperhidrosis. The benefit of dezocine is context-dependent. Optimizing satisfaction requires managing pain, autonomic side effects, and personalized analgesic selection.

## Introduction

Patient satisfaction is a cornerstone metric in modern healthcare quality assessment, directly reflecting the perceived value of care and influencing clinical outcomes, patient loyalty, and institutional reputation. As such, it serves as a robust proxy for evaluating the effectiveness of healthcare delivery systems (1–3). Surgical interventions, while therapeutic, often inflict significant tissue trauma leading to severe postoperative pain. Inadequately managed pain is a well-documented catalyst for complications such as gastrointestinal dysfunction, delayed recovery, increased healthcare utilization, and diminished patient-reported experience. Moreover, it elevates the risk of long-term sequelae including chronic postsurgical pain and postoperative depression (4–6).

Patients undergoing hepatobiliary and pancreatic surgeries represent a particularly vulnerable population. Due to the complex neuroanatomy of the upper abdomen and the extensive nature of these procedures, they frequently experience disproportionate levels of postoperative pain, even with minimally invasive techniques, necessitating proactive and potent analgesic strategies (7–9). To address this need, patient-controlled intravenous analgesia (PCIA) has become a mainstay for managing acute postoperative pain, favored for its ability to provide consistent analgesia while granting patients a sense of autonomy over their pain relief (10, 11).

Despite its advantages, the PCIA experience is not uniformly positive. A notable subset of patients reports inadequate pain relief or distressing side effects during treatment, which directly undermines their overall satisfaction with postoperative care (12, 13). Previous investigations have identified several variables, including demographic factors (e.g., age, sex, body mass index), clinical status (e.g., cancer diagnosis), and surgical characteristics, that correlate with postoperative pain intensity and analgesic consumption (10, 14–16). However, a focused analysis identifying the independent predictors of patient satisfaction specifically with PCIA following major abdominal surgery remains limited.

Therefore, this retrospective study aimed to evaluate satisfaction levels and identify the determinants of dissatisfaction among patients receiving PCIA after elective hepatobiliary and pancreatic surgery. The findings aim to inform more personalized and effective perioperative analgesic protocols, ultimately enhancing the quality of recovery.

## Methods

### Study design and setting

A retrospective analysis was conducted on data from consecutive patients who underwent elective laparotomy or laparoscopic surgery for hepatobiliary and pancreatic diseases at the First Affiliated Hospital of Zhejiang University between January 1 and December 31, 2018.

### Surgical categorization

Surgical procedures were systematically categorized based on the Modified Johns Hopkins Surgical Criteria (17, 18), which grades procedures according to technical complexity, operative duration, estimated blood loss, and anticipated physiological impact. We selected this classification system because it integrates multiple dimensions of surgical invasiveness—including anatomical extent, tissue trauma, and physiological stress—rather than relying on a single proxy such as operative time or incision length. This multidimensional approach is particularly suitable for hepatobiliary and pancreatic surgeries, which vary widely in complexity, from simple cholecystectomy to major hepatectomy and pancreaticoduodenectomy. The Modified Johns Hopkins Criteria have been previously validated in surgical outcomes research and provide a standardized framework for comparing procedures with substantially different trauma burdens. For the purpose of this study, the original five-level hierarchy was consolidated into three clinically meaningful strata (Minor, Intermediate, and Major Trauma) to facilitate statistical analysis and clinical interpretation. Specifically, Minor Trauma corresponds to Levels 1–2, Intermediate Trauma to Level 3, and Major Trauma to Levels 4–5 of the original classification. Minor Trauma procedures included cholecystectomy, endoscopic common bile duct exploration, and common bile duct exploration with T-tube drainage—procedures with limited scope and minimal tissue dissection. Intermediate Trauma included splenectomy, distal pancreatectomy, local resection of liver cancer, portal vein devascularization, segmental hepatectomy (<3 segments), and middle/distal pancreatectomy—procedures involving resection of a single organ or limited hepatic resection. Major Trauma included pancreaticoduodenectomy (Whipple procedure), proximal pancreaticoduodenectomy, total pancreatectomy, radical resection of hilar cholangiocarcinoma (Type I–III), hemihepatectomy, and major hepatectomy (≥3 segments)—procedures characterized by multi-organ resection, major vascular reconstruction, extended operative time, and significant physiological insult. This consolidated framework ensures that surgeries with comparable overall trauma burden are grouped together. When a patient underwent more than one procedure concurrently, the case was assigned to the highest corresponding trauma category based on the single most complex operation performed. This standardized approach allowed for a systematic evaluation of the relationship between surgical trauma magnitude and patient-reported outcomes, including satisfaction with postoperative analgesia (Table 1).

**Table 1:** Classification of hepatobiliary and pancreatic surgeries by Trauma severity.

Trauma severity group	Corresponding Hopkins level	Surgical procedures included	Representative examples
Minor Trauma	Levels 1–2	Procedures with limited scope, minimal tissue dissection, and short operative duration.	Cholecystectomy Endoscopic Common Bile Duct Exploration Common Bile Duct Exploration with T-tube Drainage
Intermediate Trauma	Level 3	Procedures involving resection or reconstruction of a single major organ, with moderate complexity and physiological impact.	Splenectomy Distal Pancreatectomy Local Resection of Liver Cancer Portal Vein Devascularization Segmental Hepatectomy (<3 segments) or Left Lateral Sectionectomy Middle/Distal Pancreatectomy
Major Trauma	Levels 4–5	Highly complex procedures involving multi-organ resection, major vascular reconstruction, extended operative time, and significant physiological insult.	Pancreaticoduodenectomy (Whipple Procedure) Proximal Pancreaticoduodenectomy Total Pancreatectomy Radical Resection of Hilar Cholangiocarcinoma (Type I–III) Hemihepatectomy (Right/Left Liver Resection) Major Hepatectomy (≥3 segments)

The classification is adapted from the Modified Johns Hopkins Surgical Criteria. For patients undergoing concurrent procedures, the highest trauma level among them was assigned.

### Data sources and patient selection

Data were gathered from a prospectively maintained database and electronic patient records. We excluded those who did not have PCIA pump infusion after the surgery, lacked a post-operative evaluation of acute pain service (APS) group (such as: admission to ICU or unplanned secondary surgery), and those with daily use of opioid analgesics and non-opioid adjunctive medications (e.g., gabapentin, serotonin-norepinephrine reuptake inhibitors, tricyclic antidepressants) for more than 30 days before their operation. This study was approved by the ethics committee of Zhejiang University. The written informed consent for this medical record review study was waived under the regulations of the institutional review board. The patient identification data were encoded and scrambled using a restricted computer to protect the privacy of all participants.

### Analgesia protocol and data collection

All of the surgeries were performed under general anesthesia. The pain treatments were standardized with APS treatment plan. Before the patient was sent to the post-anesthesia care unit (PACU), an electronic PCIA pump injection was set to deliver an analgesic bolus on demand. When the patient was fully awake and able to describe the pain in the surgical area, we use numerical rating scale (NRS) score (0–10; 0 = no pain, 3 = tolerable mild pain, 10 = worst pain imaginable) to evaluate their pain intensity. When the NRS score was higher than 3 we gave the patient a remedy and reassessed the pain intensity 5–10 min later until it was equal or less than 3. The parameter setting for PCIA pump was: no background dose and loading dose, bolus dose was 5–8 mL, lock-out time was 10–15 min, and maximum analgesic dose was four times bolus per hour. We instruct patient to press the analgesic pump administration button at any time according to their own needs after returning to the ward. PCIA analgesics include fentanyl (2 µg/mL) + tramadol (4 mg/mL); sufentanil (0.75 µg/mL), dezocine (0.25 mg/mL), hydromorphone (0.05 mg/mL) and oxycodone (0.15 mg/L). When patients are dissatisfied with the effect of PCIA, they will receive a selective rescue analgesia based on the patient's NRS score until the patient's NRS score is equal or lower than 3. The rescue analgesics were dezocine, oxycodone, and hydromorphone. Rescue antiemetic was used to relieve nausea and vomiting in patients.

Patient satisfaction with postoperative analgesia was evaluated by patient and recorded by APS group on the third day after surgery. The assessment was performed by the attending APS physician during a face-to-face bedside interview using a single, standardized question: “Are you satisfied with the overall effect of your postoperative pain management using the PCIA pump?” The response was recorded as a binary choice (“Satisfied” vs. “Dissatisfied”). This dichotomous approach was chosen for two reasons: first, it aligns with our institutional quality improvement protocol, which prioritizes the identification of dissatisfied patients for timely intervention; second, our preliminary data review showed that fewer than 5% of patients utilized intermediate response categories in prior pilot interviews, suggesting that a binary format minimized ambiguity in this specific clinical context. All assessments were uniformly performed on the morning of postoperative day 3 (between 8:00 AM and 10:00 AM), corresponding to the routine APS ward round schedule. This specific time point was selected because it represents a clinical equilibrium phase—sufficiently remote from the immediate post-anesthesia recovery period (postoperative day 0) to avoid confounding by residual anesthetics and emergence delirium, yet preceding the typical transition to oral analgesics and hospital discharge planning, thereby allowing the assessment to specifically reflect the patient's experience with the PCIA modality. The APS group recorded the response directly in the electronic medical record. No further granularity (e.g., Likert scale or visual analogue scale) was collected, a methodological constraint that inherently precludes analysis of intermediate satisfaction levels. At meanwhile, multidisciplinary clinical data were collected including age, gender, ASA (American Society of Anesthesiologists) score, body mass index (BMI), pain intensity at night and daytime, operation type, laparoscopy or not, PCIA analgesic, rescue analgesic or antiemetic administration, and patient negative comments. Postoperative pain intensity was described separately by the NRS score during the night and the daytime. Nausea and vomiting at any time within 72 h after surgery is classified as nausea and/or vomiting. Major postoperative complications were defined as Clavien-Dindo grade ≥ III occurring before the satisfaction assessment on postoperative day 3, and the presence of malignancy was determined based on final histopathological examination.

### Adverse effect definitions

Postoperative hyperhidrosis was defined as patient-reported profuse sweating beyond what the patient considered normal for the postoperative period, documented during the APS physician's daily bedside interviews on postoperative day 3. Patients were asked: “Have you experienced excessive sweating since your surgery?” A binary response (“Yes” or “No”) was recorded. This definition relied on patient self-report rather than clinical observation or objective measurement, consistent with the patient-centered nature of satisfaction assessment. The severity of hyperhidrosis was not graded, as no standardized grading scale was applied in the clinical setting at the time of the study. Of note, hyperhidrosis was assessed independently of pain status; patients who reported sweating were categorized as having hyperhidrosis regardless of their concurrent NRS score. This allowed us to examine whether hyperhidrosis represents a distinct adverse effect independent of uncontrolled pain, as both were recorded as separate variables in the clinical assessment.

Other adverse effects, including nausea, vomiting, dizziness, bloating, and hiccup, were assessed using the same structured patient interview and recorded as binary variables based on patient self-report (“Yes” or “No”) during the postoperative day 3 APS visit.

### Statistical analysis

Data for continuous variables were compared by the unpaired Student's *t*-test when the data were normally distributed and the Mann–Whitney *U* test when the data were not normally distributed. Categorical variables were compared by Pearson's chi-square test or Fisher's exact test. Ranking data were tested using the Mann–Whitney *U* test. Continuous variables were expressed as mean ± standard deviation (SD) or median and interquartile range (IQR). Categorical variables are expressed as numbers (%). To identify independent risk factors for patient dissatisfaction, we constructed two multivariable logistic regression models using a forced-entry (enter) method. This approach was chosen *a priori* to ensure robust adjustment for all clinically relevant confounders, irrespective of their univariate significance. Model 1 (Core Symptoms Model) included the primary patient-reported symptom predictors: postoperative pain status (NRS > 3 vs. ≤3) and hyperhidrosis. Model 2 (Extended Model) expanded upon Model 1 by further incorporating the specific PCIA analgesic regimens, the interaction term between dezocine and pain status, and a comprehensive set of clinically important covariates: age, sex, BMI, ASA physical status (I–II vs. III), surgical trauma category (Minor/Intermediate/Major), surgical approach (Open vs. Laparoscopic), presence of malignancy, and occurrence of major postoperative complications (Clavien-Dindo ≥ III). Variables with more than two categories (surgical trauma category and PCIA analgesic) were entered as dummy variables. All variables in the models were forced into the regression equation simultaneously to provide the most conservative and clinically meaningful estimates of effect. Differences were considered statistically significant if *p* < 0.05 (two-tailed). SPSS 24 (IBM Corp., Armonk, NY, USA) was used for statistical analysis and Graph Pad Prism 8.0.1 was used for graph making.

## Results

### Study cohort and patient selection

**Figure 1:**
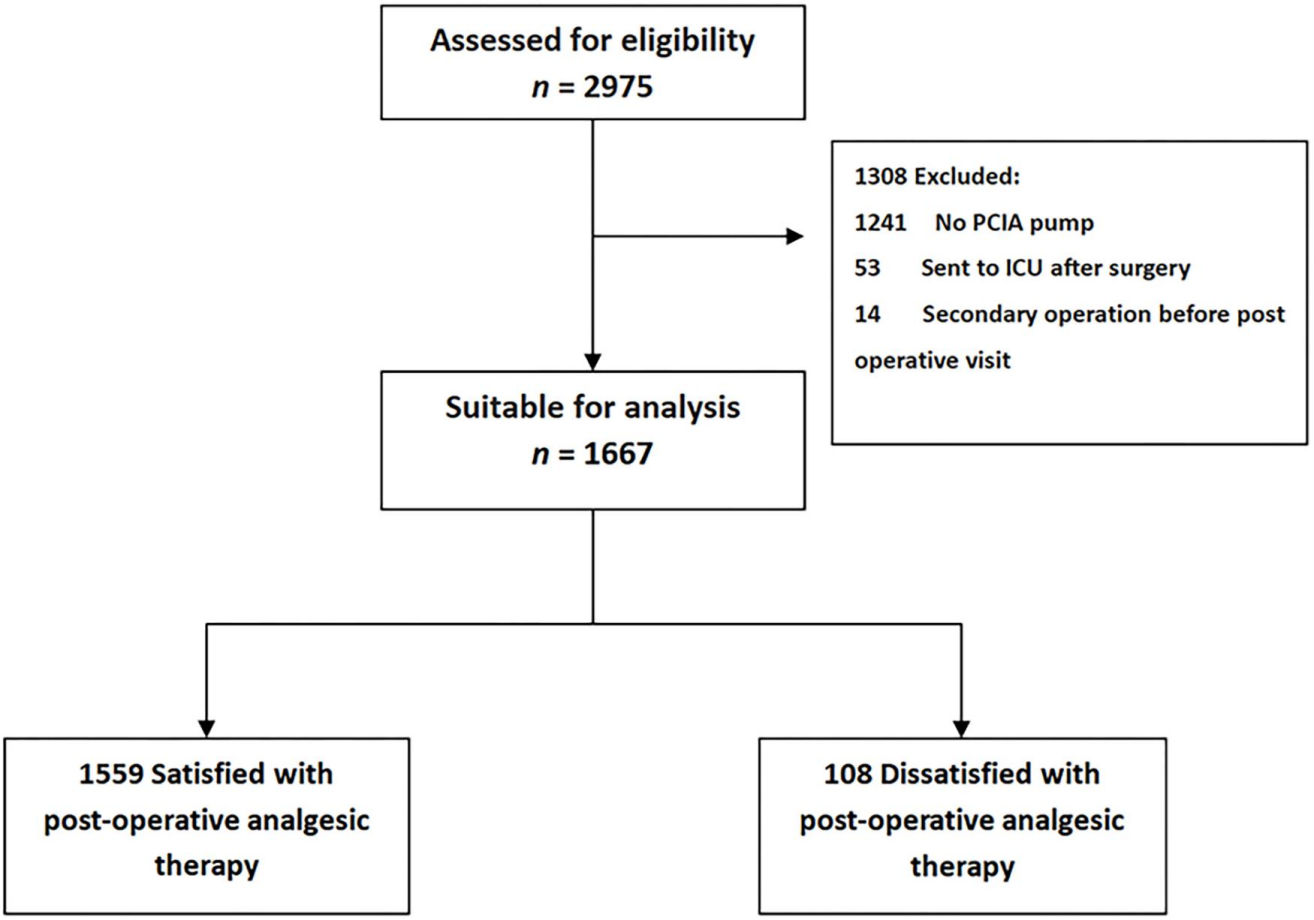
Flow diagram of patient selection and inclusion process. Of the 2,975 patients initially assessed for eligibility, 1,667 were deemed suitable for analysis. A total of 1,308 patients were excluded for the following reasons: no PCIA pump (*n* = 1,241), transfer to ICU postoperatively (*n* = 53), or requirement for a secondary operation before postoperative follow-up (*n* = 14). Among the included patients, 1,559 reported satisfaction with their postoperative analgesic therapy, while 108 expressed dissatisfaction.

Of 2,975 elective hepatopancreatobiliary surgeries performed between January 1 and December 31, 2018, 1,667 patients met the inclusion criteria for analysis following the exclusion of those without PCIA, those requiring postoperative intensive care unit (ICU) admission, or those undergoing reoperation prior to Acute Pain Service (APS) follow-up (Figure 1). Postoperative analgesia satisfaction was reported by 1,559 patients (93.5%), with 108 (6.5%) expressing dissatisfaction. The surgical distribution included 813 liver, 450 gallbladder/biliary, 339 pancreatic/pancreatoduodenal, and 65 splenic procedures. For analysis, surgeries were categorized according to their procedural complexity and associated tissue trauma (Table 1).

### Univariate and multivariable analysis of satisfaction determinants

Univariate analysis identified six variables significantly associated with dissatisfaction: gender, BMI, PCIA analgesic regimen, postoperative pain (NRS > 3), hyperhidrosis, and hiccup (all *P* < 0.05, Table 2). For the final multivariable analysis, we employed a clinically-driven, forced-entry approach, adjusting for all predefined clinically relevant confounders including age, sex, BMI, ASA status, surgical trauma category, surgical approach, presence of malignancy, and major postoperative complications, irrespective of their univariate significance.

**Table 2:** Patients’ profiles of satisfaction.

Parameter	Total	Dissatisfied	Satisfied	*P*
Total	1,667	108 (6.5%)	1,559 (93.5%)	
Age (yr)	56.6 ± 12.1	56.2 ± 1.3	56.4 ± 0.35	0.904
Gender				0.016
Female	733 (44.0%)	60 (8.2%)	673 (91.8%)	
Male	934 (56.0%)	48 (5.1%)	886 (94.9%)	
ASA				0.740
I–II	1,278 (77%)	83 (6.5%)	1,195 (93.5%)	
III	389 (23%)	25 (6.4%)	364 (93.6%)	
BMI	22.8 ± 3.3	23.3 ± 0.3	22.7 ± 0.1	0.048
Surgery type				0.186
Minor	185 (11.1%)	49 (26.5%)	136 (73.5%)	
Intermediate	1,117 (67.0%)	10 (0.9%)	1,107 (99.1%)	
Major	365 (21.9%)	49 (13.4%)	316 (86.6%)	
Laparoscopic surgery				0.121
Yes	465 (27.9%)	23 (5.0%)	442 (95.0%)	
No	1,202 (72.1%)	85 (7.1%)	1,117 (92.9%)	
Malignancy				0.621
Yes	1,021 (61.2%)	68 (6.7%)	953 (93.3%)	
No	646 (38.8%)	40 (6.2%)	606 (93.8%)	
PCIA Analgesic				0.000
Fentanyl + Tramadol	100 (6.0%)	5 (5.0%)	95 (95.0%)	
Sufentanil	231 (13.9%)	9 (3.9%)	222 (96.1%)	
Dezocine	318 (19.1%)	38 (12.0%)	280 (88.0%)	
Hydromorphone	619 (37.1%)	30 (4.9%)	589 (95.1%)	
Oxycodone	399 (23.9%)	26 (6.5%)	373 (93.5%)	
Pain		0.000		
NRS ≤ 3	1,478 (88.7%)	36 (2.4%)	1,442 (97.6%)	
NRS > 3	189 (11.3%)	72 (38.1%)	117 (61.9%)	
Nausea and/or Vomit				0.249
Yes	414 (24.8%)	32 (7.7%)	382 (92.3%)	
No	1,253 (75.2%)	76 (6.1%)	1,177 (93.9%)	
Hyperhidrosis				0.000
Yes	78 (4.7%)	16 (20.5%)	62 (79.5%)	
No	1,589 (95.3%)	92 (5.8%)	1,497 (94.2%)	
Dizziness				1.000
Yes	27 (1.6%)	1 (3.7%)	26 (96.3%)	
No	1,640 (98.4%)	107 (6.5%)	1,533 (93.5%)	
Bloating				0.522
Yes	11(%)	1 (9.1%)	10 (90.9%)	
No	1,656(%)	107 (6.5%)	1,549 (3.5%)	
Hiccup				0.004
Yes	2 (0.1%)	2 (100%)	0 (0%)	
No	1,665 (99.9%)	106 (6.4%)	1,559 (93.6%)	
Major Complications (CD ≥ III)				0.082
Yes	91 (5.5%)	10 (11.0%)	81 (89.0%)	
No	1,576 (94.5%)	98 (6.2%)	1,478 (93.8%)	

Data are presented as mean and standard deviation, or as a proportion of the total number of patients in each group. Age and BMI data were non-normally distributed and tested using the Mann–Whitney *U* test. The variables of patient sex, laparoscopic surgery, PCIA analgesic group and the major analgesia-related adverse reactions were compared by Pearson's chi-square test or Fisher's exact test. ASA and operating type are tested using the Mann–Whitney *U* test. Statistical analysis showed that there were significant differences between patients in satisfied and dissatisfied groups in terms of gender, BMI, choice of PCIA analgesic drugs, pain and the analgesia-related adverse reactions of hyperhidrosis and hiccup (*P* < 0.05).

Model 1 (Core Symptoms Model) demonstrated that moderate-to-severe postoperative pain (NRS > 3) was the strongest independent risk factor for dissatisfaction, with an aOR of 18.52 (95% CI: 11.20–30.60, *P* < 0.001), indicating an 18.5-fold increase in the odds of dissatisfaction. Hyperhidrosis was also identified as a strong independent risk factor, with an aOR of 6.29 (95% CI: 3.05–13.89, *P* < 0.001), indicating a 6.3-fold increase in the odds of dissatisfaction. The model demonstrated good discriminatory ability (AUC = 0.845, 95% CI: 0.799–0.891) and acceptable calibration (Hosmer–Lemeshow *P* = 0.110).

Model 2 (Extended Model) further incorporated the specific PCIA analgesic regimens and the interaction terms between each analgesic and postoperative pain status. In this extended model, pain remained a strong independent risk factor but with a slightly attenuated effect (aOR = 14.93, 95% CI: 9.52–26.32, *P* < 0.001), while hyperhidrosis retained a nearly identical effect (aOR = 6.29, 95% CI: 3.05–13.89, *P* < 0.001). The attenuation of the pain effect in Model 2 suggests that a portion of the pain-related dissatisfaction is explained by the differential efficacy of analgesic regimens across pain strata. The extended model showed improved discriminative ability (AUC = 0.879, 95% CI: 0.840–0.917) and acceptable calibration (Hosmer–Lemeshow *P* = 0.180).

  *T*he logistic regression equation for Model 1 was:

Ln[(P/(1−P)])=f(x)=3.845–3.246pain−1.632hyperhidrosis[−othercovariates−]

For Model 2, incorporating analgesic-specific effects and interactions:

Ln[(P/(1−P)])=f(x)=3.872–2.696pain−1.839hyperhidrosis+0.286PCIAanalgesic_sufentanil+0.243PCIAanalgesic_dezocine+0.066PCIAanalgesic_hydromorphone−0.350PCIAanalgesic_oxycodone−2.034dezocine_pain[−othercovariates−]

The analysis revealed a significant interaction effect between dezocine and postoperative pain status (interaction *P* < 0.001), indicating that the impact of dezocine on patient satisfaction critically depended on the patient's postoperative pain level. Stratified analysis of the dezocine effect (Table 3) revealed: Among patients with mild or no pain (NRS ≤ 3), dezocine showed no significant advantage or disadvantage compared with the reference regimen (fentanyl + tramadol): aOR = 1.22 (95% CI: 0.33–4.68, *P* = 0.771). Among patients with moderate to severe pain (NRS > 3), dezocine was associated with an ∼87% reduction in the odds of dissatisfaction compared with the reference regimen: aOR = 0.18 (95% CI: 0.06–0.55). This protective effect was statistically significant (*P* < 0.001). No similar statistically significant interactions were observed for sufentanil, hydromorphone, or oxycodone (all interaction *P* > 0.05), suggesting that the context-dependent benefit is unique to dezocine among the analgesics assessed in this study.

**Table 3:** Stratified effect of dezocine on patient satisfaction according to postoperative pain status.

Pain status	Comparison	Adjusted odds ratio (aOR)*	95% confidence interval (CI)	*P* value	Interpretation
Mild/no pain (NRS ≤ 3)	Dezocine vs. fentanyl + tramadol (Reference)	1.22	0.33–4.68	0.771	No significant difference in satisfaction.
Moderate/severe pain (NRS > 3)	Dezocine vs. fentanyl + tramadol (Reference)	0.18	Calculated: 0.06–0.55^a^	<0.001^‡^	∼87% reduction in odds of dissatisfaction.

aORs are derived from the multivariate logistic regression model (Model 2) adjusting for hyperhidrosis, all other analgesic regimens, age, sex, BMI, ASA status, surgical trauma category, surgical approach, presence of malignancy, and major postoperative complications.

a The 95% CI for the aOR in the Moderate/Severe Pain group (0.17) was calculated based on the variance-covariance matrix of the coefficients for Dezocine and the Dezocine × Pain interaction term.

‡ The *P* value for the comparison in the Moderate/Severe Pain group is derived from the significance of the Dezocine × Pain interaction term (*P* < 0.001), which directly tests the difference in dezocine's effect between pain strata.

### Comparison of predictive model performance

We compared the two multivariable logistic regression models to elucidate factors influencing satisfaction (Table 4, Supplementary Figure 1). Model 1 (Core Symptoms Model), incorporating pain and hyperhidrosis as the primary patient-reported symptom predictors and adjusted for all clinically relevant confounders, demonstrated good discriminative ability (AUC = 0.845) and confirmed that both moderate-to-severe postoperative pain (aOR = 18.52) and hyperhidrosis (aOR = 6.29) were strong, independent risk factors for dissatisfaction. Model 2 (Extended Model), which additionally incorporated the specific analgesic regimens and the dezocine  ×  pain interaction term, yielded a significant improvement in predictive performance, with the AUC increasing to 0.879 (AUC difference = 0.034, *P* for comparison < 0.01). The Nagelkerke *R*^2^ also improved from 0.334 to 0.372, indicating that the addition of analgesic-specific variables explained additional variance in patient satisfaction beyond that captured by symptoms alone. The Hosmer–Lemeshow goodness-of-fit test demonstrated acceptable calibration for both models (*P* = 0.110 and *P* = 0.180 for Models 1 and 2, respectively), supporting the reliability of the identified predictors. The robust discriminative capacity (AUC > 0.84) of both models further validates that our findings are not driven by overfitting or case-mix, but represent genuine associations between pain, side effects, analgesic choice, and patient satisfaction.

**Table 4:** Multivariable logistic regression analyses for predictors of patient dissatisfaction with postoperative PCIA.

Predictor	Model 1 (core symptoms model)			Model 2 (extended model)		
	aOR	95% CI	*P* value	aOR	95% CI	*P* value
Postoperative pain (NRS > 3 vs. ≤3)	18.52	11.20–30.60	<0.001	14.93	9.52–26.32	<0.001
Hyperhidrosis (Yes vs. No)	6.29	3.05–13.89	<0.001	6.29	3.05–13.89	<0.001
Dezocine × Pain (Interaction term)	—	—	—	0.13	0.04–0.43	<0.001
PCIA Analgesic (Ref: Fentanyl + Tramadol)						
Sufentanil	—	—	—	1.33	0.39–4.53	0.648
Dezocine	—	—	—	1.28	0.33–4.91	0.724
Hydromorphone	—	—	—	1.07	0.37–3.09	0.903
Oxycodone	—	—	—	0.71	0.24–2.07	0.523
Age (per year)	1.01	0.99–1.03	0.45	1.010	0.99–1.03	0.380
Sex (Male vs. Female)	0.97	0.60–1.58	0.90	1.000	0.61–1.62	0.989
BMI (per kg/m²)	0.98	0.91–1.05	0.530	0.98	0.91–1.05	0.520
ASA (III vs. I–II)	1.09	0.63–1.88	0.752	1.06	0.61–1.84	0.834
Surgical Trauma (Ref: Minor)						
Intermediate	1.20	0.52–2.76	0.670	1.25	0.54–2.90	0.604
Major	1.22	0.70–2.13	0.492	1.19	0.68–2.08	0.543
Surgical approach (lap vs. open)	0.86	0.50–1.48	0.580	0.88	0.51–1.52	0.650
Malignancy (Yes vs. No)	0.890	0.55–1.45	0.634	0.91	0.56–1.49	0.710
Major complications (CD ≥ III) (Yes vs. No)	1.72	0.79–3.77	0.172	1.70	0.77–3.75	0.188
Model performance	AUC = 0.845 (0.799–0.891) Nagelkerke *R*^2^ = 0.334 HL *P* = 0.003			AUC = 0.879 (0.840–0.917) Nagelkerke *R*^2^ = 0.372 HL *P* = 0.04		

Both models were constructed using a forced-entry (enter) method. Model 1 (Core Symptoms Model) included patient-reported symptom predictors (pain, hyperhidrosis) and was adjusted for all covariates listed. Model 2 (Extended Model) incorporated these symptoms plus the specific analgesic regimens, the dezocine × pain interaction, and the same panel of covariates. The reference category for analgesics was fentanyl combined with tramadol. The Hosmer–Lemeshow test *P*-values are now non-significant, indicating acceptable model calibration after the inclusion of all relevant covariates. Model performance was assessed by the area under the receiver operating characteristic curve (AUC), Nagelkerke's pseudo *R*^2^, and the Hosmer–Lemeshow goodness-of-fit test.

### Analysis of adverse effect distribution

**Figure 2:**
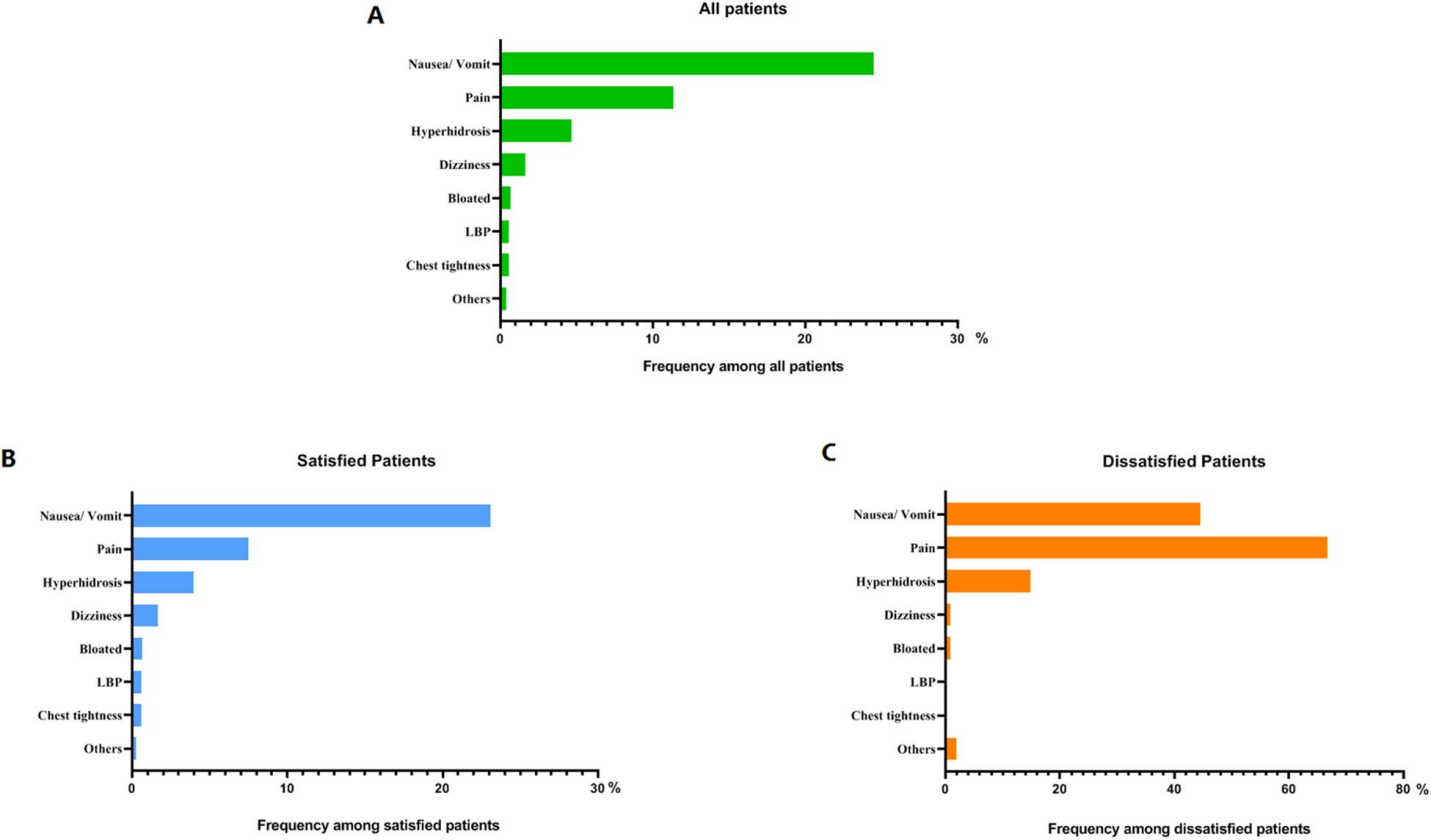
Negative feedback on postoperative analgesia in patients. We analyzed patient-reported adverse effects following postoperative analgesia. Among all negative evaluations, nausea and/or vomiting, pain, hyperhidrosis, and dizziness were the four most frequently reported symptoms, accounting for approximately 95% of all complaints. In the overall patient population **(A)** and among patients satisfied with analgesia **(B)**, the most common adverse reaction was nausea and/or vomiting. In contrast, pain was the predominant adverse reaction reported by patients dissatisfied with analgesia **(C)** Statistically significant differences were observed between the satisfied and dissatisfied groups in the incidence of pain (*P* < 0.001), hyperhidrosis (*P* < 0.001), and hiccups (*P* < 0.01).

Based on the categorization of patient satisfaction with postoperative analgesia, the distribution of adverse reactions was systematically summarized (Figure 2). Nausea and/or vomiting, pain, hyperhidrosis, and dizziness constituted the majority of negative evaluations during the 72-h postoperative analgesia period, collectively representing approximately 95% of all reported complaints. Among all patients (Panel A) and those satisfied with analgesia (Panel B), nausea and/or vomiting was the most frequently recorded adverse effect. In contrast, pain emerged as the predominant complaint in the dissatisfied group (Panel C). Comparative analysis further revealed that the incidence of pain, hyperhidrosis, and hiccups differed significantly between the satisfied and dissatisfied cohorts (all *P* < 0.01).

### Association between PCIA pump discontinuation and satisfaction

Patient satisfaction with postoperative analgesia was significantly associated with the decision to discontinue the PCIA pump in response to adverse reactions. Among patients who experienced adverse analgesic effects, approximately 81.06% opted to stop the pump, and this group reported significantly higher satisfaction scores compared to those who continued pump use (*P* = 0.0022, Supplementary Figure 2). Those who stopped using the pump expressed markedly higher satisfaction compared with those who continued. Within the pump continued group, satisfaction rates were only 31.25% among patients who reported no pain and 25% among those who experienced pain.

### Patterns of postoperative pain and dissatisfaction

**Figure 3:**
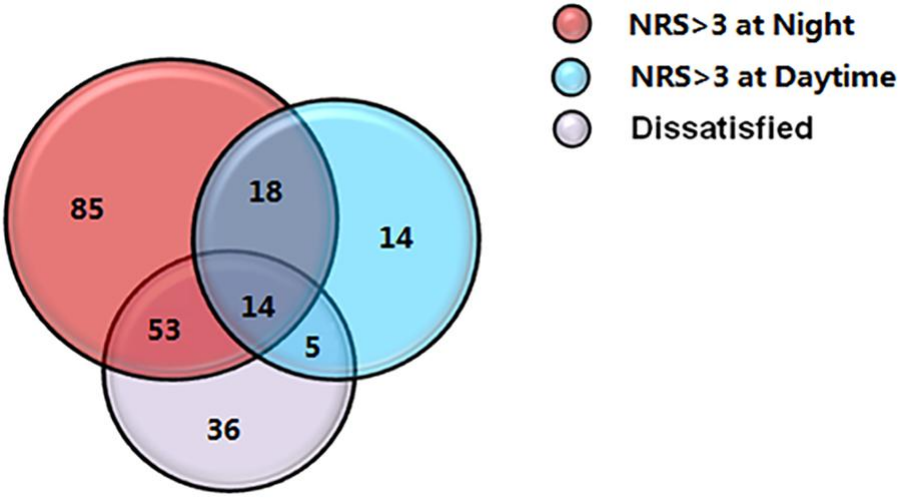
Distribution of patient dissatisfaction according to postoperative pain patterns. Among patients with pain only on the night of surgery (NRS > 3), 53 of 138 (38.41%) were dissatisfied with analgesia. For those with pain only on the second day, 5 of 19 (26.32%) expressed dissatisfaction. Of the 32 patients who experienced pain on both the night of surgery and the following day, 14 (43.75%) were dissatisfied. In contrast, among the 1,478 patients without significant postoperative pain, 36 (2.44%) reported dissatisfaction due to other analgesic-related reasons.

The association between patterns of postoperative pain and patient dissatisfaction with analgesia is summarized in Figure 3. Dissatisfaction was most prevalent among patients who experienced pain on both the night of surgery and the following day, with 14 of 32 (43.75%) expressing dissatisfaction. For patients with pain confined to the night of surgery, 53 of 138 (38.41%) reported dissatisfaction. A lower rate was observed in those with pain only on the second day (5 of 19, 26.32%). In contrast, among patients without significant postoperative pain, dissatisfaction due to other analgesia-related reasons was minimal, affecting only 36 of 1,478 patients (2.44%).

Postoperative pain intensity significantly differed between patients satisfied and dissatisfied with analgesia (Supplementary Table 1). The incidence of pain both at night and during the daytime was markedly higher in the dissatisfied group compared to the satisfied group (all *P* < 0.01). Within the dissatisfied cohort, nighttime pain alone was the most prevalent pattern, reported in 49.07% of these patients, which was substantially higher than the rates of daytime-only pain (4.63%) or pain at both time points (7.41%) (*P* < 0.001).

### Subgroup analysis of hyperhidrosis

Postoperative hyperhidrosis was observed in 78 of 1,667 patients (4.7%). Compared to patients without hyperhidrosis, those with hyperhidrosis underwent major surgery more frequently (35.9% vs. 21.2%, *P* = 0.002) and received different compositions of PCIA analgesics (*P* = 0.024), with a higher proportion using sufentanil (23.1% vs. 13.4%). The use of rescue analgesics was also more common in the hyperhidrosis group (2.6% vs. 0.3%, *P* = 0.029). No significant differences were found in gender, age, BMI, ASA classification, prevalence of laparoscopy, incidence of significant pain (NRS > 3), nausea/vomiting, or low blood pressure between the two groups (all *P* > 0.05) (Supplementary Table 2).

In the multivariable logistic regression analysis adjusted for age, sex, BMI, ASA status, surgical trauma category, surgical approach, and PCIA analgesic type, rescue analgesic use was identified as an independent risk factor for postoperative hyperhidrosis (OR = 8.82, 95% CI 1.49–52.20, *P* = 0.016). Compared to minor surgery, intermediate surgery was associated with a reduced likelihood of hyperhidrosis (OR = 0.48, 95% CI 0.24–0.95, *P* = 0.035), while major surgery did not show a statistically significant association. No significant differences in the incidence of hyperhidrosis were observed across different PCIA analgesic regimens (overall *P* = 0.022) (Supplementary Table 3).

### Comparison of perioperative and hospitalization outcomes

No statistically significant differences were found between satisfied and dissatisfied patients regarding perioperative and hospitalization outcomes after adjusting for baseline and surgical covariates. Preoperative fasting time, rates of surgery-related complications and unplanned reoperations, postoperative and total hospital stay durations, total medical costs, and readmission rates within 3 months of discharge were all comparable between the two groups (all *P* > 0.05, Supplementary Table 4).

## Discussion

This retrospective cohort study elucidates the multifactorial nature of patient dissatisfaction with PCIA following major hepatobiliary and pancreatic surgery (12, 19). Our analysis establishes that dissatisfaction is not a simple reflection of analgesic failure, but rather a composite outcome shaped by the interplay of unrelieved symptoms, specific adverse effects, pharmacological agent selection, and perioperative care dynamics. The robustness of these findings is strengthened by our comprehensive adjustment for a wide array of clinically relevant confounders, including patient demographics, comorbidities, surgical complexity, and major postoperative complications, ensuring that the identified predictors are independent of these underlying factors.

### Core symptom burden: pain and hyperhidrosis as primary drivers

The foremost independent predictors of dissatisfaction were moderate-to-severe postoperative pain (NRS > 3) and profuse sweating (hyperhidrosis). In Model 1, Patients with NRS > 3 had a nearly 18.52-fold increase in the odds of dissatisfaction (aOR = 14.93, 95% CI: 9.52–26.32, *P* < 0.001), and those with hyperhidrosis had a 6.29-fold increase in the odds of dissatisfaction (aOR = 6.29, 95% CI: 3.05–13.89, *P* < 0.001). In Model 2, which additionally accounted for analgesic regimens and the dezocine–pain interaction, the pain effect was slightly attenuated but remained substantial (aOR = 14.93, 95% CI: 9.52–26.32, *P* < 0.001), while hyperhidrosis retained a nearly identical effect (aOR = 6.29, 95% CI: 3.05–13.89, *P* < 0.001). This attenuation of the pain effect in Model 2 suggests that a portion of the pain-related dissatisfaction is explained by the differential efficacy of analgesic regimens—particularly dezocine—across pain strata. The potency of severe pain in undermining satisfaction is well-documented, as it is directly linked to suffering, prolonged recovery, and increased healthcare utilization (4, 14, 20). Hyperhidrosis, however, emerged with particular salience in our cohort. While often regarded as a minor opioid side effect, its strong independent association with dissatisfaction suggests it represents a significant source of patient distress. This symptom likely serves as a clinical marker of autonomic nervous system hyperactivity, which can be triggered by either inadequately controlled pain (sympathetic activation) or by opioid therapy itself (e.g., through histamine release or other mechanisms) (21–23). Notably, the literature on postoperative hyperhidrosis is sparse outside the context of compensatory sweating after sympathectomy (24), highlighting a gap our study begins to address.

### The conditional efficacy of dezocine: clinical finding and pharmacological rationale

A pivotal and novel finding is the significant interaction between the analgesic dezocine and postoperative pain severity. Dezocine's effect was strictly context-dependent: it provided no measurable satisfaction benefit over standard therapy (fentanyl plus tramadol) in patients with mild pain (NRS ≤ 3) (aOR = 1.22, 95% CI: 0.33–4.68, *P* = 0.771), but it conferred a substantial protective effect against dissatisfaction in those with moderate-to-severe pain (NRS > 3) (aOR = 0.18, 95% CI: 0.06–0.55), corresponding to an approximately 87% reduction in the odds of dissatisfaction. However, this finding should be interpreted with caution: the wide confidence interval reflects limited precision due to the small subgroup of patients with both NRS > 3 and dezocine exposure, and retrospective interaction analyses are susceptible to residual confounding. Therefore, while biologically plausible, this hypothesis-generating finding requires prospective validation before clinical implementation. This conditional efficacy finds a plausible explanation in dezocine's unique receptor pharmacology as a mixed κ-opioid agonist and μ-opioid partial agonist (25, 26). In high-pain states, where central sensitization and heightened autonomic drive are prominent, the *κ*-agonist component may offer complementary analgesic pathways, while the μ-partial agonist activity may provide a more favorable side-effect profile. Unlike pure μ-agonists (e.g., fentanyl, sufentanil), which can cause dose-dependent autonomic disturbances including sweating (27), partial agonists exhibit a “ceiling effect” that might limit such dysregulation (28). Thus, dezocine may uniquely balance potent analgesia with mitigated autonomic side effects specifically when pain burden is high, a hypothesis directly supported by our statistical interaction model.

### Beyond pain scores: the Complex construct of satisfaction

Our results reinforce that satisfaction is imperfectly correlated with pain scores alone. A subset of patients reported high satisfaction despite NRS > 3, a paradox observed in other studies (29–35). This may be attributable to patients valuing transient pain relief, perceived attentiveness of care, or meeting pre-set expectations more than the peak pain intensity itself (29, 32, 33). We found supportive evidence: satisfaction was significantly higher in patients whose significant pain (NRS > 3) occurred only during daytime, when access to rescue medication and clinical staff is greater, compared to those with nocturnal or persistent pain.

This day-night disparity underscores another critical dimension. Nocturnal pain was associated with disproportionately higher dissatisfaction, despite similar recorded pain scores. This likely results from a “double burden”: reduced nursing presence and intervention delays at night, coupled with the circadian amplification of pain perception mediated by hormones like melatonin and cortisol (36–38). Furthermore, the management of side effects proved crucial. Interestingly, patients who discontinued PCIA due to adverse reactions maintained high satisfaction (>94%), likely viewing it as a regaining of control. In stark contrast, satisfaction plummeted (25%–31%) in those who endured side effects without stopping therapy. This indicates that the perceived ability to manage one's treatment trajectory is a key component of satisfaction.

### Hyperhidrosis: a multifactorial side effect warranting scrutiny

The role of hyperhidrosis warrants deeper discussion. Beyond being a mere predictor, its etiology in our surgical context appeared pharmacologically dominant. After controlling for ambient temperature, it was independently associated with both the class of PCIA analgesic and the magnitude of surgery. The higher incidence with sufentanil and fentanyl-tramadol regimens points toward serotonergic/noradrenergic mechanisms, as tramadol and some metabolites of potent opioids can inhibit neurotransmitter reuptake (39, 40). The potent link between hyperhidrosis and the need for rescue analgesia (OR = 8.82) further suggests it may be a physiological marker of poorly controlled pain or incipient opioid-induced hyperalgesia (23, 27). For clinical management, our findings suggest that strategies like opioid rotation, use of transdermal fentanyl, or adjunctive agents such as antihistamines or scopolamine (41–43) could be considered when problematic sweating arises.

### Model performance and clinical implications

The evolution from the core symptoms model (AUC = 0.845) to the institutional analgesics model (AUC = 0.879) is instructive. While patient-reported symptoms form the foundation, incorporating specific therapeutic choices significantly improves predictive power. This underscores that institutional protocols and personalized analgesic selection are actionable levers for improving outcomes. Although goodness-of-fit tests indicated room for model refinement, the high discriminative capacity validates the robustness of the identified predictors. The consistent performance of both models after rigorous adjustment for multiple confounders confirms that our findings are not driven by case-mix or surgical complexity, but represent genuine associations between pain, side effects, analgesic choice, and satisfaction.

### Limitations

Several methodological limitations should be considered when interpreting our findings. First, this was a single-center retrospective study, which inherently limits causal inference and may introduce unmeasured confounding, particularly from psychological status, preoperative pain history, and chronic pain or psychiatric illness. Second, although we evaluated analgesic types, detailed opioid exposure data—including total opioid consumption, number of PCIA demands, delivered doses, and rescue analgesic frequency—were not available in our dataset. Without these granular dose-related variables, it remains difficult to determine whether patient dissatisfaction is primarily attributable to drug selection, inadequate dosing, or their combined effects. Third, we excluded patients admitted to the ICU postoperatively (*n* = 53), as they lacked standardized APS evaluation. These excluded patients likely represented a subset with the highest surgical trauma and pain burden; their exclusion may have led to underestimation of dissatisfaction rates and limits the generalizability of our findings to higher-acuity patients. Our results are therefore most directly applicable to patients with uncomplicated postoperative courses managed on general wards. Fourth, satisfaction was assessed as a binary outcome using a single non-validated question rather than a multi-dimensional instrument (e.g., APS-POQ-R, QoR-15); additionally, the exact wording of the satisfaction question was not standardized across the study period, which may have introduced measurement variability, and inter-rater reliability was not formally assessed. Fifth, all satisfaction evaluations were fixed to the morning of postoperative day 3, which may introduce recall bias and fail to capture the dynamic nature of satisfaction. Sixth, the generalizability of our findings may be further constrained by institutional-specific analgesic protocols and the limited use of dezocine in contemporary practice, as analgesic practices have evolved considerably since 2018. Seventh, important clinical variables that could influence satisfaction—including operative duration, estimated blood loss, intraoperative opioid administration, regional analgesia use, cancer stage, ICU stay after APS assessment, and surgeon variability—were not available for adjustment, and residual confounding from these omitted factors is therefore highly likely.

Despite these limitations, the large sample size, standardized data collection, and robust discriminatory power of our predictive models (AUC > 0.84) support the reliability of the identified predictors. Future prospective multicenter studies with validated satisfaction instruments, serial postoperative assessments, comprehensive opioid dosing records, inclusion of higher-acuity patients, and detailed perioperative data collection are warranted to validate and extend our conclusions.

### Future directions

Future prospective, multicenter studies are needed to validate these findings and to explore the mechanisms linking autonomic markers (e.g., sweating) to analgesic dissatisfaction. Research should also investigate the cost-effectiveness and side-effect profiles of dezocine compared with other opioids in high-risk patient subgroups. Moreover, greater attention should be directed toward comprehensively evaluating perioperative factors—including analgesic dosing, surgical stress parameters, and psychosocial variables—to better understand and improve postoperative pain management and patient satisfaction.

## Conclusion

In conclusion, dissatisfaction with PCIA is a multifaceted outcome primarily driven by severe pain and the distressing symptom of hyperhidrosis. The context-dependent benefit of dezocine highlights the potential of mechanism-based analgesic selection, particularly for patients with high postoperative pain. Ultimately, optimizing satisfaction requires a paradigm that looks beyond pain scores to proactively manage autonomic side effects, respect circadian and personal control factors, and tailor analgesic therapy to the individual patient's physiological and experiential state.

## Data Availability

The original contributions presented in the study are included in the article/Supplementary Material, further inquiries can be directed to the corresponding authors.
