# Supplementary material for: Patient satisfaction with postoperative analgesia after hepatobiliary and pancreatic surgery: a retrospective analysis
Source: Front Surg. 2026 Jul 28;13:1887173. doi: 10.3389/fsurg.2026.1887173 (PMC13457289; doi:10.3389/fsurg.2026.1887173)
Supplement: Supplementary file 1 [file Table1.docx]

**Supplementary Table 1. Distribution and comparison of postoperative pain intensity between satisfaction groups.**

| Parameter | Total | Dissatisfied108 | Satisfied1559 | *P* |
| --- | --- | --- | --- | --- |
| NRS>3 at Night | 138 (10.98%) | 53(49.07%) | 85 (5.45%) | 0.000 |
| NRS>3 at Both | 32 (1.92%) | 14 (7.41%) | 18 (1.16%) | 0.000 |
| NRS>3 at Daytime | 19 (1.14%) | 5 (4.63%) | 14 (0.90%) | 0.006 |

Patients in the dissatisfied group exhibited significantly higher incidences of pain both at night and during the daytime compared to those in the satisfied group (all P < 0.01). Within the dissatisfied group, the proportion of patients experiencing only nighttime pain (49.07%) was substantially higher than those with only daytime pain (4.63%) or pain at both time points (7.41%) (P < 0.001).
