# Supplementary material for: Patient satisfaction with postoperative analgesia after hepatobiliary and pancreatic surgery: a retrospective analysis
Source: Front Surg. 2026 Jul 28;13:1887173. doi: 10.3389/fsurg.2026.1887173 (PMC13457289; doi:10.3389/fsurg.2026.1887173)
Supplement: Supplementary file 2 [file Table2.docx]

**Supplementary Table 2. Baseline Characteristics and Perioperative Variables Stratified by the Presence of Postoperative Hyperhidrosis.**

|  | Hyperhidrosis | | *P* |
| --- | --- | --- | --- |
|  | Yes | No |  |
| Total | 78 | 1589 |  |
| Gender (Female) | 36 (46.2%) | 697 (43.9%) | 0.716 |
| Age | 57 (52, 66) | 57 (49,65) | 0.898 |
| BMI | 22.0 (19.5, 24.4) | 22.6 (20.6, 24.8) | 0.118 |
| ASA ≤ 2 | 54 (69.2%) | 1224 (77.0%) | 0.131 |
| Operation Type |  |  | 0.002 |
| Minor Surgery | 12 (15.4%) | 173 (10.9%) |  |
| Intermediate Surgery | 38 (48.7%) | 1079 (67.9%) |  |
| Major Surgery | 28(35.9%) | 337 (21.2%) |  |
| Laparoscopy | 22 (28.2%) | 443 (27.9%) | 1.000 |
| PCIA Analgesics |  |  | 0.024 |
| Fentanyl + Tramadol | 5 (6.4%) | 95 (6.0%) |  |
| Sufentanil | 18 (23.1%) | 213 (13.4%) |  |
| Dezocine | 11 (14.1%) | 307 (19.3%) |  |
| Hydromorphone | 34 (43.6%) | 585 (36.8%) |  |
| Oxycodone | 10 (12.8%) | 389 (24.5%) |  |
| Rescue Analgesic | 2 (2.6%) | 4 (0.3%) | 0.029 |
| NRS ＞ 3 | 30 (38.5%) | 493 (31.0%) | 0.171 |
| Nausea and/or Vomit | 24 (30.8%) | 390 (24.5%) | 0.227 |
| Low BP | 1 (1.3%) | 8 (0.5%) | 0.351 |

Data are presented as n (%) for categorical variables and median (interquartile range) for continuous variables. P values were derived from the chi-square test (or Fisher’s exact test where appropriate) for categorical variables and the Mann–Whitney U test for continuous variables.
