# Supplementary material for: Patient satisfaction with postoperative analgesia after hepatobiliary and pancreatic surgery: a retrospective analysis
Source: Front Surg. 2026 Jul 28;13:1887173. doi: 10.3389/fsurg.2026.1887173 (PMC13457289; doi:10.3389/fsurg.2026.1887173)
Supplement: Supplementary file 3 [file Table3.docx]

**Supplementary Table 3. Factors associated with postoperative hyperhidrosis: multivariable logistic regression analysis.**

| Predictor | OR | 95% CI | | *P* Value |
| --- | --- | --- | --- | --- |
|  |  | Lower | Upper |  |
| Analgesic  (reference: Fentanyle+ Tramadol) |  |  |  | 0.022 |
| Sufentanil | 1.931 | 0.680 | 5.482 | 0.216 |
| Dezocine | 0.735 | 0.240 | 2.245 | 0.588 |
| Hydromorphone | 1.232 | 0.462 | 3.287 | 0.676 |
| Oxycodone | 0.564 | 0.185 | 1.720 | 0.314 |
| Operation Type  (reference: Minor) |  |  |  | 0.005 |
| Medium | 0.476 | 0.238 | 0.950 | 0.035 |
| Major | 1.052 | 0.504 | 2.195 | 0.892 |
| Rescue Analgesic | 8.815 | 1.488 | 52.204 | 0.016 |

Rescue analgesic use was an independent risk factor for postoperative hyperhidrosis (OR = 8.82, 95% CI 1.49–52.20, *P* = 0.016). Compared with minor surgery, intermediate surgery was associated with a lower likelihood of hyperhidrosis (OR = 0.48, 95% CI 0.24–0.95, *P* = 0.035), while major surgery showed no significant association. The type of PCIA analgesic was not significantly associated with the occurrence of hyperhidrosis (overall *P* = 0.022).
