# Supplementary material for: Patient satisfaction with postoperative analgesia after hepatobiliary and pancreatic surgery: a retrospective analysis
Source: Front Surg. 2026 Jul 28;13:1887173. doi: 10.3389/fsurg.2026.1887173 (PMC13457289; doi:10.3389/fsurg.2026.1887173)
Supplement: Supplementary file 4 [file Table4.docx]

**Supplementary Table 4. Comparison of perioperative and hospitalization outcomes between patients satisfied and dissatisfied with postoperative analgesia.**

| Parameter | Patient Satisfaction | | *P* |
| --- | --- | --- | --- |
|  | Satisfied | Dissatisfied |  |
| Fasting Time | 4.57±3.78 | 4.42±3.22 | 0.881 |
| Surgery-related Complications | 105 (6.7%) | 10 (9.3%) | 0.317 |
| Unplanned Reoperation | 50 (3.2%) | 6 (5.6%) | 0.190 |
| Post-operative Hospital Stay | 11.64±9.42 | 11.36±8.27 | 0.894 |
| Total Length of Hospitalization | 20.74±11.64 | 20.88±10.61 | 0.556 |
| Total Medical Cost | 8.27±14.64 | 7.61±4.87 | 0.444 |
| Re-admission within three months of discharge | 55 (3.5%) | 4 (3.7%) | 0.924 |

No significant differences were observed between the two groups in terms of preoperative fasting time, incidence of surgery-related complications, unplanned reoperation rates, postoperative or total hospital stay length, total medical cost, or readmission rate within three months after discharge (all *P* > 0.05).
